# Prevalence and time trends of overweight, obesity and severe obesity in 447,925 Swedish adults, 1995–2017

**DOI:** 10.1177/1403494820914802

**Published:** 2020-04-30

**Authors:** Erik Hemmingsson, Örjan Ekblom, Lena V. Kallings, Gunnar Andersson, Peter Wallin, Jonas Söderling, Victoria Blom, Björn Ekblom, Elin Ekblom-Bak

**Affiliations:** 1 Åstrand Laboratory of Work Physiology, The Swedish School of Sport and Health Sciences, Stockholm, Sweden; 2 Health Profile Institute, Stockholm, Sweden; 3 Department of Medicine, Karolinska Institutet, Stockholm, Sweden

**Keywords:** Overweight, obesity, severe obesity, prevalence, time trends, Sweden, adults

## Abstract

*Aims*: The purpose of this research was to describe the current prevalence and historic trends in overweight, obesity and severe obesity in Sweden. *Methods*: Data on BMI, age, gender, education and geographic region were obtained in *n*=447,925 Swedish adults through a nationwide screening test (1995–2017). To account for sampling variations, we quantified prevalence estimates and time trends using standardized values (direct method) to all 18–74-year-old Swedes, using nationwide databases. Rates of overweight (BMI ⩾25 kg/m^2^), obesity (BMI ⩾30 kg/m^2^) and severe obesity (BMI ⩾35 kg/m^2^) were calculated across gender, age, education and geographic categories. Years were grouped into two-year sampling periods (except the first period where we used three years) for increased power. We used multivariable logistic regression to quantify independent associations between age, gender, education and region with obesity development and current prevalence rates. *Results*: In 2016/17 the unstandardized prevalence of overweight, obesity and severe obesity were 55.1%, 16.6% and 4.2%, respectively. Factors associated with a higher obesity prevalence were male gender, older age, lower education and residing in a rural region (all *P*<0.001). Between 1995 and 2017 the prevalence of severe obesity increased by 153%, compared to obesity (+86%) and overweight (+23%). While there were similar increases in obesity across gender and age groups, people with low education (vs high) and rural areas (vs urban) had a higher prevalence increase (both *P*<0.001). ***Conclusions*: Rates of overweight, obesity and severe obesity have increased markedly in Swedish adults since 1995. Priority groups for prevention efforts include individuals with low education and those living in rural areas**.

## Background

Obesity is a major threat to public health through its association with many of our leading causes of morbidity and mortality [1,2]. Obesity is also associated with huge costs to society through increased health care expenditures, sick leave and reduced productivity [3], and individuals with obesity suffer pervasive stigmatization and discrimination [4].

There are clear indications that most Western societies experience greater increases of obesity in lower socioeconomic position (SEP) groups compared to high SEP groups, and that this effect may be increasing [5–7]. Having a low SEP has been identified as a primary upstream driver of obesity, for example through influences on factors such as increased stress, low grade inflammation and unhealthy lifestyle choices [8].

Previous limitations in studies on obesity prevalence and time trends in Sweden include self-report data (primarily height and weight), which tend to provide biased results (underestimations), as well as having samples that may include an insufficient number of individuals across important obesity prognostic variables (gender, education, socioeconomic position, etc.) to allow for a detailed assessment of how obesity rates are developing across different segments of the population [9–12].

## Aims

We aimed to describe the current (2016–2017) prevalence estimate of obesity (BMI ⩾30 kg/m^2^) and severe obesity (BMI ⩾35 kg/m^2^) using objective data in a large cohort of Swedish adults, with accompanying time trends in obesity and severe obesity between 1995 and 2017. Estimates and trends were mapped across categories of gender, age, education and geographic region.

## Methods

### Recruitment

This study used data from the Health Profile Assessment (HPA) database [13,14], managed by the HPI Health Profile Institute (Stockholm, Sweden), which was responsible for the standardization of methods and education of data collection staff. Participation was optional and free of charge for the individual and was offered to all employees working for a company or organization connected to occupational or other health services.

From October 1982 until May 2017, a total of 519,152 participants (18 to 74 years old) were registered and stored in a central database. The annual inclusion rate was substantially lower in the formative years (1982: *n*=1, 1994: *n*=888), compared to more recent years (1995: *n*=1347, 2016: *n*=34,177). We therefore restricted our analysis to include only the years 1995 to 2017 (*n*=522,381). Of these, *n*=447,925 provided valid data of all needed variables (see below).

### Data overview

The Health Profile Assessment is an interdisciplinary method comprised of three components: (a) a questionnaire with data on current lifestyles, previous and current physical activity habits, perceived health and overall stress; (b) an in-depth interview with data on age, gender, marital status and occupation; (c) anthropometry testing with data on body weight and height. Body mass was assessed with a calibrated scale in light-weight clothing to the nearest 0.5 kg. Body height was measured to the nearest 0.5 cm using a stadiometer. Highest obtained educational level and place of residence were obtained by linking the personal identity number with data from Statistics Sweden.

The complete Health Profile Assessment data set, comprising *n*=520,831 individuals from 1995 to June 2017, was missing key data in the following cases: age (*n*=295), education (*n*=2934), BMI (*n*=69,677), leaving *n*=447,925 for the final analysis.

### Study design

The participants (*n*=447,925) were consecutively recruited to the Health Profile Assessment database between 1995 and 2017. All data are cross-sectional. In order to perform trend analyses, we grouped all years into two-year periods (except the first period, where we used three years) for reduced sampling variations and increased power: 1995–1997, 1998–1999, 2000–2001, 2002–2003,

2004–2005, 2006–2007, 2008–2009, 2010–2011, 2012–2013, 2014–2015, 2016–2017. Each sampling period contained unique individuals, that is, there were no prospective data. In cases where individuals had data from more than one time period, only data from the first measurement were used. In order to compare obesity rates and trends across sampling periods, across important obesity prognostic variables (age, gender, education, geographic region), we quantified time trends using standardized values (direct method) to the entire population of 18–74 year olds in Sweden in 2015 (*n*=6,842,976), using data from Statistics Sweden (a national database).

### Statistics

Standardized mean prevalence rates of overweight (BMI ⩾25 kg/m^2^), obesity (BMI ⩾30 kg/m^2^) and severe obesity (BMI ⩾35 kg/m^2^) were calculated both overall and across categories of gender, age (18–34 years, 35–49 years, 50–74 years), education (<9y, 10–12y, ⩾12y) and county (rural, urban or mixed) for all time periods. The most recent time period (2016/2017) was used to calculate the current prevalence estimate.

The mean prevalence rates were standardized to the population in Sweden in 2015 using sex, age (18–24y, 25–34y, 35–44y, 45–49y, 50–54y, 55–64y, 65–74y), and length of education (<9y, 10–12y, ⩾12y). In the years 1995–2001, the study cohort included few individuals aged 65–74 years old (*n*<5) across strata of sex and education, while 5–10% of the population in Sweden in 2015 was in the age group of 65–74 years, resulting in few individuals contributing with unreasonable large weights. For this reason, we excluded observations in strata of sex, age and education including <5 individuals with a weight of >5% (*n*=13 observations were excluded).

We used multivariable logistic regression to quantify independent associations between age, gender, education and region with obesity development and current prevalence rates. Possible interactions between sub-groups (gender, age, education, geographic region) per year increase in odds ratio for overweight, obesity and severe obesity, respectively, were studied using the procedure described by Altman and Bland [15].

## Results

The proportion of men and women varied across sampling periods, with increasingly more men participating in Health Profile Assessments, see Table I. Participation rates by age group (18–34, 35–49 and 50–74 years) were similar across sampling periods, but with a continuous trend of more participants with high education (>12 years) and fewer with low education levels (⩽9 years) from 1995–1997 to 2016–2017.

**Table 1.** Participant characteristics across the different sampling periods. Data are mean (SD), unless otherwise stated.

	**n**	**Age (Y)**	**% Men**	**Body weight (kg)**	**Height (m)**	**BMI (kg/m** ^2^ **)**	**Education** **n (%)**			**Geography** **n (%)**		
							Low	Middle	High	Rural	Mixed	Urban
**1995-1997**	5 380	41.2 (10.1)	49%	75.3 (14.1)	1.73 (0.09)	25.1 (3.8)	2854 (53%)	1800 (33%)	726 (13%)	2575 (48%)	1703 (32%)	1102 21%)
**1998-1999**	8 092	42.4 (10.4)	55%	76.5 (14.2)	1.74 (0.09)	25.2 (3.8)	3775 (47%)	2770 (34%)	1547 (19%)	3052 (38%)	2492 31%)	2548 (32%)
**2000-2001**	15 807	42.9 (10.9)	51%	76.6 (14.6)	1.73 (0.09)	25.4 (3.9)	6796 (43%)	5776 (37%)	3236 (20%)	3986 (25%)	6002 (38%)	5819 (37%)
**2002-2003**	27 531	42.9 (11.4)	47%	76.0 (14.8)	1.73 (0.09)	25.4 (4.0)	11705 (43%)	10460 38%)	5367 (19%)	7180 (26%)	8336 (30%)	12015 (44%)
**2004-2005**	46 549	43.8 (11.1)	48%	76.7 (15.0)	1.73 (0.09)	25.5 (4.1)	18272 (39%)	16887 (36%)	11393 (24%)	11623 (25%)	15954 (34%)	18972 (41%)
**2006-2007**	47 317	43.7 (11.3)	52%	77.7 (15.4)	1.74 (0.09)	25.7 (4.2)	18176 (39%)	17866 (38%)	11280 (24%)	9338 (20%)	15926 34%)	22053 (47%)
**2008-2009**	52 007	43.5 (11.5)	53%	78.4 (15.8)	1.74 (0.09)	25.8 (4.3)	19444 (37%)	19348 (37%)	13219 25%)	8921 (17%)	14767 (28%)	28319 (55%)
**2010-2011**	45 299	43.0 (11.4)	56%	79.2 (16.0)	1.74 (0.09)	26.0 (4.4)	16148 (36%)	17106 (38%)	12051 (27%)	6519 (14%)	14795 (33%)	23985 (53%)
**2012-2013**	79 160	43.0 (11.5)	61%	79.8 (15.9)	1.75 (0.09)	26.0 (4.3)	24338 (30%)	30978 (39%)	23850 (30%)	11690 (15%)	23515 (30%)	43955 (56%)
**2014-2015**	73 682	42.4 (11.7)	63%	80.6 (16.3)	1.75 (0.09)	26.1 (4.4)	21893 (30%)	30819 42%)	20975 (28%)	12937 (18%)	22458 (31%)	38287 (52%)
**2016-2017**	47 069*	41.7 (11.9)	64%	80.9 (16.5)	1.75 (0.09)	26.2 (4.4)	12994 (28%)	20501 (44%)	13574 (29%)	6202 (31%)	9062 28%)	17158 (53%)

* *n* = 32 782 for Geography.

### Prevalence of overweight, obesity and severe obesity in 2016–2017

The prevalence of overweight (BMI ⩾25 kg/m^2^), obesity (BMI ⩾30 kg/m^2^) and severe obesity (BMI ⩾35 kg/m^2^) were 55.1%, 16.6% and 4.2%, respectively. Men had significantly higher rates of overweight (62.7% vs 41.7%; adj. OR: 2.24, 95% CI: 2.15–2.33) and obesity (18.1% vs 14.4%, adj. OR: 1.18, 95% CI: 1.12–1.25) compared to women but lower rates of severe obesity (4.2% vs 4.3%, adj. OR: 0.82, 95% CI: 0.75–0.91), after adjustment for covariates (see Table II).

**Table II.** Prevalence of overweight, obesity and severe obesity in Swedish adults during 2016–2017 (*n*=47,069, unstandardized data).

	**BMI ⩾25 kg/m** ^2^		**BMI ⩾30 kg/m** ^2^		**BMI ⩾35 kg/m** ^2^	
	%	OR (95% CI)	%	OR (95% CI)	%	OR (95% CI)
**All**	55.1%		16.6%		4.2%	
Women (*n*=17,130)	41.7%	1 (ref)	14.4%	1 (ref)	4.3%	1 (ref)
Men (*n*=29,939)	62.7%	2.24 (2.15–2.33)	18.1%	1.18 (1.12–1.25)	4.2%	0.82 (0.75–0.91)
**Age**						
18–34 y (*n*=14,785)	43.6%	1 (ref)	11.8%	1 (ref)	3.3%	1 (ref)
35–49 y (*n*=18,451)	56.6%	1.82 (1.74–1.91)	17.6%	1.57 (1.47–1.67)	4.7%	1.37 (1.22–1.54)
50–74 y (*n*=13,833)	65.3%	2.29 (2.18–2.42)	20.5%	1.61 (1.50–1.73)	4.7%	1.16 (1.02–1.32)
**Education**						
<9 y (*n*=12,994)	69.4%	2.26 (2.14–2.39)	24.5%	2.70 (2.51–2.91)	6.3%	3.15 (2.73–3.63)
9–12 y (*n*=20,501)	54.9%	1.64 (1.56–1.71)	16.3%	1.90 (1.77–2.03)	4.3%	2.18 (1.90–2.49)
>=12 y (*n*=13,574)	41.7%	1 (ref)	9.6%	1 (ref)	2.2%	1 (ref)
**Region** ^a^						
Rural (*n*=6202)	61.5%	1.37 (1.29–1.46)	21.0%	1.49 (1.38–1.61)	5.6%	1.68 (1.46–1.93)
Mixed (*n*=9062)	58.4%	1.17 (1.10–1.23)	18.3%	1.23 (1.15–1.33)	5.1%	1.48 (1.31–1.69)
Urban (*n*=17,518)	50.9%	1 (ref)	13.8%	1 (ref)	3.2%	1 (ref)

a Data on region were missing in *n*=14,287 participants (those recruited during 2017).

OR for gender, age and education were mutually adjusted, but not geographic region due to missing data (see note above).

OR for region were fully adjusted (covariates: gender, age and education).

Younger individuals had lower rates of overweight, obesity and severe obesity vs older individuals (*P*<0.001 for all), see Table II. Similarly, we found a consistent pattern of higher prevalence rates of overweight, obesity and severe obesity in people with low education (vs high education) and in people residing in rural (vs urban) areas.

### Time trends in overweight, obesity and severe obesity, 1995–2017

**Figure 1.**
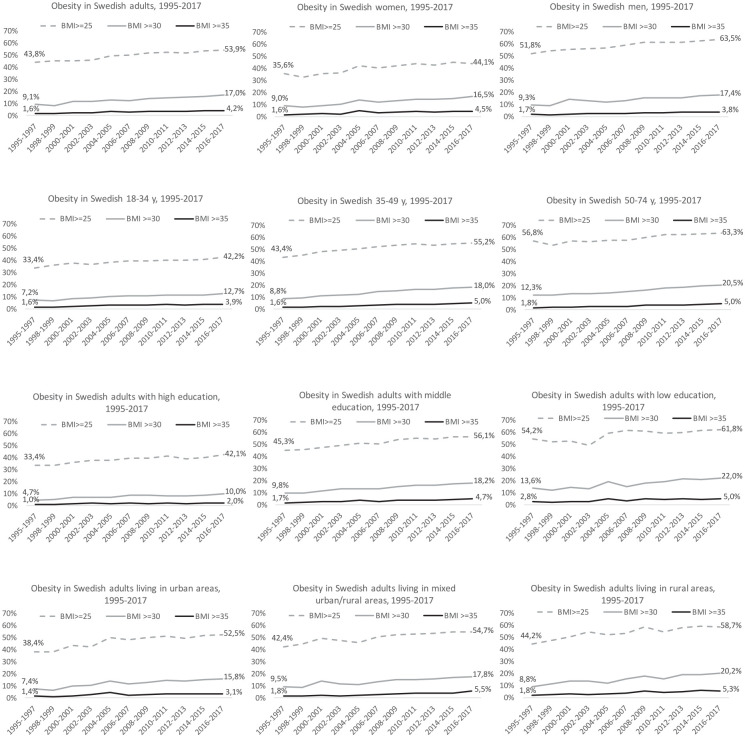
Time trends in prevalence of overweight (BMI: ⩾25 kg/m^2^), obesity (BMI: ⩾30 kg/m^2^) and severe obesity (BMI: ⩾35 kg/m^2^) in Swedish adults (*n*=447,925), across gender, age categories, education levels and degree of urbanization, 1995–2017. All data are standardized across time periods.

From 1995–2017, the mean BMI increased from 25.1 kg/m^2^ to 26.2 kg/m^2^, corresponding to 5.6 kg in body weight. The prevalence of overweight increased from 43.8% to 53.9%, obesity increased from 9.1% to 17.0%, and severe obesity increased from 1.6% to 4.2%, see Figure 1. The rates of overweight, obesity and severe obesity increased across all categories of gender, age, education level and geographical region. We noted higher growth rates for severe obesity (+153%) than obesity (+86%) and overweight (+23%). Men and women experienced the same growth pattern for all BMI categories, see Table III. Only middle-aged (35–49 years) individuals had a greater increase in obesity compared to the other age groups. People with low education (vs high) and those living in rural areas (vs urban) had more pronounced growth across all BMI categories.

**Table III.** Odds ratios (95% CI) for changes in weight status category (BMI⩾25 kg/m^2^, BMI⩾30 kg/m^2^ and BMI⩾35 kg/m^2^) between 1995 and 2017, in the total population and across sub-groups (*n*=447,925, standardized data).

	**BMI ⩾25 kg/m** ^2^	**BMI ⩾30 kg/m** ^2^	**BMI ⩾35 kg/m** ^2^
	OR (95% CI)	OR (95% CI)	OR (95% CI)
**Total sample**	1.022 (1.021–1.023)	1.038 (1.036–1.040)	1.052 (1.048–1.055)
**Gender**			
Men	1.022 (1.020–1.024)	1.037 (1.034–1.040)	1.048 (1.042–1.053)
Women	1.022 (1.020–1.023)	1.038 (1.036–1.041)	1.055 (1.050–1.060)
	*P*=1.00	*P*=0.62	*P*=0.07
**Age**			
18–34 years	1.025 (1.022–1.027)	1.039 (1.035–1.044)	1.050 (1.042–1.058)
35–49 years	1.024 (1.022–1.026)	1.043 (1.040–1.046)	1.057 (1.051–1.062)
⩾50 years	1.023 (1.020–1.025)	1.037 (1.034–1.040)	1.054 (1.047–1.060)
	18–34 vs 35–49, *P*=0.54 18–34 vs ⩾50, *P*=0.27 34–49 vs ⩾50, *P*=0.54	18–34 vs 35–49, *P*=0.15 18–34 vs ⩾50, *P*=0.47 34–49 vs ⩾50, *P*=0.006	18–34 vs 35–49, *P*=0.16 18–34 vs ⩾50, *P*=0.45 34–49 vs ⩾50, *P*=0.49
**Education**			
Low	1.024 (1.022–1.026)	1.038 (1.036–1.041)	1.052 (1.047–1.057)
Middle	1.026 (1.024–1.028)	1.042 (1.039–1.046)	1.057 (1.051–1.064)
High	1.012 (1.009–1.014)	1.030 (1.025–1.034)	1.036 (1.026–1.046)
	Low vs Middle, *P*=0.17 Low vs High, *P*<0.001 Middle vs High, *P*<0.001	Low vs Middle, *P*=0.07 Low vs High, *P*=0.002 Middle vs High, *P*<0.001	Low vs Middle, *P*=0.23 Low vs High, *P*=0.005 Middle vs High, *P*=0.001
**Region**, *n*=433,606			
Rural	1.026 (1.023–1.029)	1.042 (1.039–1.046)	1.058 (1.051–1.064)
Mixed	1.028 (1.026–1.031)	1.046 (1.043–1.049)	1.066 (1.059–1.073)
Urban	1.021 (1.019–1.023)	1.036 (1.033–1.039)	1.045 (1.039–1.051)
	Rural vs Mixed, *P*=0.32 Rural vs Urban, *P*=0.007 Mixed vs Urban, *P*<0.001	Rural vs Mixed, P=0.09 Rural vs Urban, *P*=0.01 Mixed vs Urban, *P*<0.001	Rural vs Mixed, *P*=0.10 Rural vs Urban, *P*=0.004 Mixed vs Urban, *P*<0.001

* Data on region were missing in *n*=14,319 participants (those recruited during 2017).

OR for changes in weight status across categories of gender, age and education were mutually adjusted, but not geographic region due to missing data (see foot note above).

OR for region were fully adjusted (covariates: gender, age and education).

While the prevalence of severe obesity was much lower than obesity and overweight during 1995–2017 (see Figure 1), the growth rates for severe obesity were consistently higher than the corresponding increases in obesity and overweight across all prognostic categories (gender, age, education, geographic region).

## Discussion

### Main findings

In this large cohort (*n*=447,925) of Swedish adults, with objective measurements of BMI spanning 23 years (1995–2017), the rates of overweight, obesity and severe obesity increased across all categories of gender, age, education level and geographic region. The prevalence of overweight, obesity and severe obesity (54%, 17% and 4%, respectively) were comparatively low from a European perspective [16–18].

There were, however, no discernible signs that obesity rates were stabilizing or reversing during this 23-year period. The increase in severe obesity (+153%) was alarming, with an 86% increase in obesity and 24% increase in overweight. A similar difference in growth pattern across BMI categories (i.e. strongest growth for severe obesity, compared to obesity and overweight) was noticed across all prognostic categories (gender, age, education, geographic region). Education and geographic region were two particularly important prognostic variables, highlighting a greater need to prevent further obesity growth in individuals with lower education and in people living in rural areas.

### Previous studies

Other prevalence studies of Swedish adults show a fairly consistent pattern of increasing BMI and corresponding rates of obesity and severe obesity, although arguably slightly lower estimates than in the current study [11,16]. This may be due to this study using objective measures of body weight and height, as opposed to self-reported values, which are associated with underestimations, but also because those studies only included data up to 2010 [11].

The findings of this study also corroborate that the highest overall growth rates for the different BMI categories were seen for severe obesity and obesity, whereas rates of overweight tend to be more stable [19,20]. This suggests that many obesity-prone individuals are continuing their upward weight trajectory, and that over time they develop more severe forms of obesity. The pattern of higher obesity rates in men than women has also been noted previously, as have higher rates among less well-educated individuals [18,21].

### Implications for public health

The continued increase in obesity prevalence, particularly severe obesity, must be considered a failure of public health policy [2,22]. Our findings, therefore, have several implications for policy makers. The question of obesity prevention has been on the public health agenda for several decades in Sweden, although there has been a dearth of policy measures to counter the epidemic. While neighbouring countries such as Norway, Finland and Denmark have introduced active policies to counter obesity, Sweden has yet to implement any wide-ranging anti-obesity policy measures.

It is important to recognize that obesity, and particularly severe obesity, are still very resistant to conventional treatment, that is, lifestyle changes [23–25]. This highlights a much greater role for prevention, particularly for infants and young children, that is, the time period when adult adipocyte quantity and body weight trajectories are largely determined [26,27]. Meaningful prevention therefore needs to focus on young families and children, providing ample opportunities for healthy and balanced nutrition and physical activity, as well as a stable family environment [8]. From an adult perspective, it is urgent to find measures that help individuals lead more balanced lifestyles to at least prevent further weight gain, especially among low socioeconomic position groups [28,29].

### Strengths and limitations

Limitations include a sample consisting of employed adults, meaning that the sample was likely biased towards individuals with higher education and higher socioeconomic position. We were not able to quantify the proportion of individuals in public or private employment, or to ascertain whether our sample otherwise differed from the overall population, for example in terms of health investment. But given the inverse association between obesity and socioeconomic position [7], this suggests that our findings may be slightly underestimated. Since there were few observations before 1995, we were not able to caption the development of obesity in Sweden since its inception around the early to mid 1980s. While the current cohort cannot claim to be representative of all of adults living in Sweden, its large size and inclusion of several important demographic factors nevertheless allowed us to quantify the considerable heterogeneity in how obesity has developed across multiple groups in society.

Strengths include a large sample, which allowed us to quantify trends across different categories of age, gender, education and location with adequate statistical power. The data collection period spanned 23 years and included objective data on body weight and height (as opposed to self-report, which tends to be biased). We were also able to use nationwide databases for access to data on obtained education level, as well as using the same databases for standardizing the values from each sampling period, meaning that our findings were unlikely to be biased by yearly variations in the gender, age, education and geographic region of the included individuals.

## Conclusion

Between 1995 and 2017 there was a steady increase in adult obesity (BMI ⩾30 kg/m^2^, from 9.1% to 17.0%) and severe obesity (BMI ⩾30 kg/m^2^, 1.6% to 4.2%) in Sweden. While this development was consistently seen across age groups, in men and women, in all adult age categories, as well as in rural and urban areas, we noted larger prevalence increases in people with lower vs higher education and in those residing in rural vs urban areas. Efforts to prevent obesity are urgently needed.
